# The History of Colistin Resistance Mechanisms in Bacteria: Progress and Challenges

**DOI:** 10.3390/microorganisms9020442

**Published:** 2021-02-20

**Authors:** Mouna Hamel, Jean-Marc Rolain, Sophie Alexandra Baron

**Affiliations:** 1 IRD, APHM, MEPHI, Faculté de Médecine et de Pharmacie, Aix Marseille University, CEDEX 05, 13385 Marseille, France; hamel.mouna@gmail.com (M.H.); jean-marc.rolain@univ-amu.fr (J.-M.R.); 2 IHU Méditerranée Infection, CEDEX 05, 13385 Marseille, France

**Keywords:** polymyxins, colistin history, mechanisms of action and resistance, technological approaches, innovative technologies

## Abstract

Since 2015, the discovery of colistin resistance genes has been limited to the characterization of new mobile colistin resistance (*mcr*) gene variants. However, given the complexity of the mechanisms involved, there are many colistin-resistant bacterial strains whose mechanism remains unknown and whose exploitation requires complementary technologies. In this review, through the history of colistin, we underline the methods used over the last decades, both old and recent, to facilitate the discovery of the main colistin resistance mechanisms and how new technological approaches may help to improve the rapid and efficient exploration of new target genes. To accomplish this, a systematic search was carried out via PubMed and Google Scholar on published data concerning polymyxin resistance from 1950 to 2020 using terms most related to colistin. This review first explores the history of the discovery of the mechanisms of action and resistance to colistin, based on the technologies deployed. Then we focus on the most advanced technologies used, such as MALDI-TOF-MS, high throughput sequencing or the genetic toolbox. Finally, we outline promising new approaches, such as omics tools and CRISPR-Cas9, as well as the challenges they face. Much has been achieved since the discovery of polymyxins, through several innovative technologies. Nevertheless, colistin resistance mechanisms remains very complex.

## 1. Introduction

Over the last 70 years, the polymyxin family of antibiotics, including polymyxin B and colistin (also called polymyxin E), has experienced an uncommon fate. The polymyxins were initially considered “miracle” antibiotics when they were first commercialized in the 1950s, with a bactericidal efficacy against Gram-negative bacteria (GNB) and a low level of resistance [1]. Colistin was subsequently abandoned in the 1980s in favour of other less toxic broad-spectrum antibiotics before regaining the forefront in the 2000s for the treatment of multidrug-resistant GNB infections [2]. As a result, the pharmacokinetic (PK) and pharmacodynamic (PD) properties, as well as the resistance mechanisms developed by the target bacteria, remain poorly understood [3]. Several studies deciphered the mechanism of action of colistin without being able to elucidate it completely in the 1950s. At that time, polymyxin resistance was revealed by the detection of in vitro resistant mutants [4]. Moreover, 30 years of clinical disuse resulted in a lack of knowledge of the minimum inhibitory concentration (MIC) determination in vitro and its optimal use in the clinic [3]. In 2007, the reclassification of polymyxins as a major agent for the treatment of multidrug-resistant GNB infections by the World Health Organization (WHO) revived interest in clinical research on this antibiotic [1]. Consequently, data on PK/PD were collected, and new resistance mechanisms were elucidated. The discovery of the first transferable colistin resistance gene in 2015, the *mcr-1* gene (for *mobile colistin resistance* gene), is the most significant example. This finding highlighted the major role of the animal reservoir in the transmission and diffusion of this antibiotic-resistance gene [5]. In fact, colistin has been used for many years in veterinary medicine as a growth factor and in the prophylaxis and treatment of livestock infections [3]. Investigations into colistin resistance mechanisms revealed the complexity of the pathways by which bacteria defend themselves against colistin activity [2]. It appears that the first targets identified as being responsible for colistin resistance were not sufficient to explain resistance in every isolate, suggesting the existence of other mechanisms involved in polymyxin resistance [1].

New technological tools have become available for researchers over the last few years and have generated a multitude of data to analyse. The remaining challenge is to understand and analyse such information in order to identify new pathways involved in colistin resistance. The aim of this review, through the scientific and clinical history of colistin, is to identify technological methods and interesting targets currently responsible for colistin resistance. In this review, we will resituate the current knowledge of colistin resistance mechanisms by the methods used for their discovery. We will then focus on the contribution of genomics in increasing our knowledge of colistin resistance and on the issues raised by genomic analysis and the limitations of this method. Proteome analysis is a method currently used to answer these questions, and its advantages and limitations will be detailed. Finally, we will discuss the benefits of new genetic tools such as the CRISPR-Cas9 technique, and how it might be useful in this field.

## 2. Pathways Leading to Colistin Action and Mechanisms of Resistance 

### 2.1. Mode of Action

**Figure 1:**
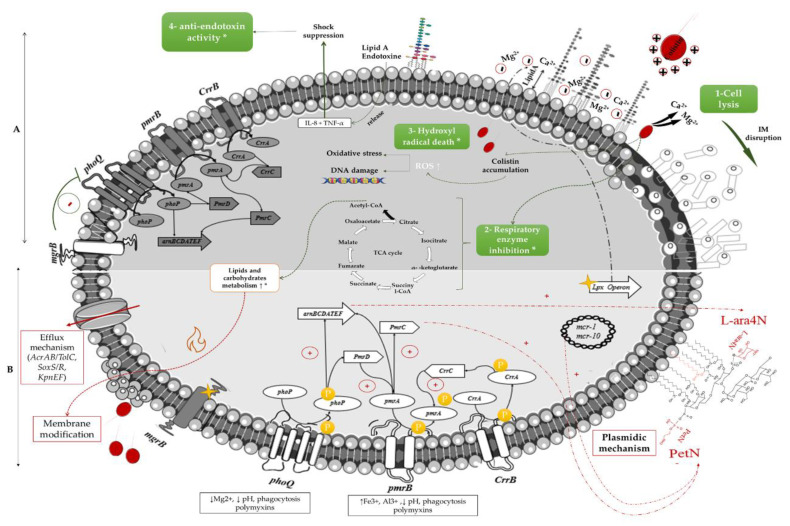
Mechanisms of action and resistance to colistin in Gram-negative organisms. (**A**): mechanisms of action of colistin, (**B**): colistin-resistance mechanisms, * on respiratory enzyme inhibition, Hydroxyl radical death, anti-endotoxin activity and the metabolism of lipids and carbohydrates represent the recently described mechanisms of action and resistance, which remain unclear so far; The yellow stars represent mutations resulting in the inactivation of regulatory genes. Green arrows indicate the mechanism of action for a susceptible strain, the red arrows indicate the different pathways involved in colistin resistance, the grey shades demonstrate the differences in the gene expression between the mechanism of action and resistance

Colistin is an old polypeptide antibiotic of the group E, discovered in 1947 by Y. Koyama from *Paenibacillus polymyxa* subspecies *colistinus* cultures [6]. It is a bactericidal, narrow-spectrum molecule directed against most GNB, but ineffective against Gram-positive bacteria, anaerobic bacteria, and mycoplasmas [3]. The main target of the polymyxins is the lipopolysaccharide (LPS) of GNB membranes [1]. The lipid A of the outer part of the LPS is negatively charged and interacts with divalent cations (mainly Mg^2+^ and Ca^2+^), allowing an overall stabilization of the outer membrane [2]. Colistin, a net positively charged molecule, has therefore a strong affinity to bind to the LPS, leading to a displacement of cations by electrostatic interaction. It results in a disorganization of the membrane structure, with release of the LPS [1]. Colistin is then introduced into the outer membrane through its lipophilic acid-fat chain. Colistin alters the permeability of the outer membrane, allowing it to insert itself and reach the inner membrane. A disorganization of this inner membrane then occurs by breaking the integrity of the phospholipid bilayer [7]. Eventually, lysis of this membrane results in the release of intracellular contents and death of the bacteria (Figure 1). This process is the most commonly used mechanism to explain the antibacterial action of colistin, but the ultimate mechanisms leading to cell death are still not well understood [7].

Other potential mechanisms of action have been identified, such as vesicle-vesicle contact. After colistin crosses the outer membrane, lipid exchanges between the inner and outer membrane take place, causing structural changes in the membranes with a loss of phospholipids, leading to an osmotic imbalance that lyses the bacteria [8]. The accumulation of free radicals linked to the oxidative stress induced by colistin is also a pathway responsible for DNA, protein, and lipid damage, leading to bacterial death. Inhibition of vital respiratory enzymes and endotoxin activity of lipid A have been described as well. Endotoxin activity involves the inhibition of this activity of lipid A of LPS by colistin, by binding to LPS molecules and neutralizing them, resulting in the release of tumour necrosis factor-alpha (TNF-a) and Interleukin 8 (IL-8) cytokines and thus the suppression of the shock. [7] (Figure 1).

### 2.2. Mechanisms of Resistance

The most common mechanism of colistin resistance is due to chromosomal mutation in genes associated with the modification of the lipid A of LPS, the primary target of colistin, as an adaptative strategy [2]. Such alterations can be obtained by the addition of phosphoethanolamine (PEtN) and 4-amino-4-deoxy-L-arabinose (L-Ara4N) to the phosphate groups of lipid A [1]. The genes that encode enzymes involved in lipid A synthesis are *pmrHFIJFKLM* (also known as *arnBCADTEF-pmrE*). These genes are up-regulated by the two-component systems (TCS) PmrAB and PhoPQ, the latter being negatively regulated by the *mgrB* gene [9] (Figure 1). Other strategies include the use of efflux pumps and capsule formation [1]. Moreover, the horizontal transfer of the plasmid-carried gene *mcr-1* encoding for PEtN has become an important cause of the spread of polymyxin resistance among various GNB [5]. The origin of *mcr* enzymes can be retraced back to the 1980s in China [10].

In the following, we will focus on the methods that enabled discovery of these major mechanisms and on the methods that are currently of the greatest interest in enabling us to understand the unresolved mechanisms.

## 3. From Electron Microscopy to the Discovery of Regulatory Genes

Electron microscopy was the first tool used to explain colistin’s mechanism of action, demonstrating morphologic changes such as loss of nuclear material and granularity of the cytoplasm [11]. Observations in *Escherichia coli* and *Pseudomonas aeruginosa* demonstrated that treatment with polymyxin B or E modifies the cell wall, forming cell wall projections or “blebs” and the release of cytoplasmic contents through splits in the cell envelope [12]. Their presence grew with the polymyxin concentration, and they were inhibited by magnesium ions [13]. Using a fluorescent method, Newton et al. demonstrated in 1954 that Mg^2+^ and other divalent cations antagonized the activity of polymyxin B at a negatively charged site on *P. aeruginosa* [14].

Several other studies examined this phenomenon using alternative techniques as evidence that they were not artefacts. Schindler and Teuber examined *Salmonella typhimurium* treated with polymyxin B by freeze-etching, a less destructive technique for the cell envelope [15]. This technique revealed three layers, which were interpreted as the outer and inner monolayer of the outer membrane and the outer surface of the cytoplasmic membrane. The formation of numerous blebs extended only to the outer monolayer of the outer membranes [15]. It was demonstrated that these particles were outer membrane fragments with a phospholipid/lipopolysaccharide/protein ratio similar to that of the outer membranes [13]. The researchers compared polymyxins to cationic detergents, in that they are charged similarly at neutral pH, since detergents disorganize the cell membrane and denature certain proteins. They suggested that polymyxin bactericidal activity is due to its ability to combine with bacterial cell structures and disorganize them, leading to loss of the cell’s osmotic balance [16]. Based on this, little information was available concerning the mechanism by which polymyxin E exerts its bactericidal action. To elucidate it, lipid extraction procedures and chromatography showed that polymyxins not only bind to these lipids but also significantly alter their structures [17].

Polymyxins rapidly became the treatment of first choice for GNB infections, as it was supposed that one of the characteristics of polymyxins was bacterial difficulty in developing resistance, and that resistant strains were unstable and do not easily occur in vitro; bacteria become easily susceptible in the absence of polymyxins [18]. Later, facultative resistance to colistin from a genetic trait emerged; homologous recombination tests have shown that it was due to frequent reverse mutations that vary from species to species. The selection media seems to play a role in maintaining the frequency of mutants [4]. Conrad et al. showed that culture of *P. aeruginosa* on different media containing various sources of carbon affects the sensitivity of the cells to polymyxins. An adaptive resistance was observed when the carbon source was D-glucose or L-glutamate, and the latter was also associated with alterations in unsaturated fatty acids [19]. Thus, the mechanism for the development of polymyxin B resistance has been shown to be one of metabolic readjustment and media-dependent [20].

### 3.1. Intrinsic Resistance Bacteria

Studies of strains naturally resistant to colistin contributed to the understanding of colistin activity and the basic mechanisms of colistin resistance. *Proteus* species and *Serratia marcescens* are among the *Enterobacteriaceae* and are regularly resistant to the action of polymyxins [21,22]. The extraction and chromatography of lipids from a wild type *Proteus sp*., highly resistant to polymyxin B, and its polymyxin B-sensitive mutant, demonstrated that phospholipid compositions were nearly similar. Each organism contained similar amounts of N-methyl-phosphatidylethanolamine, in addition to similar amounts of phosphatidylethanolamine, phosphatidylglycerol, and cardiolipin [22]. This finding demonstrates that the composition of the lipid of the envelope was not responsible, but rather the cell envelope, which restricts polymyxin access to sensitive lipid target sites [22]. Douglas et al. employed electron microscopy to observe the effect of polymyxin B on the polysaccharide components of the outer membrane in *S. marcescens*. A dense, dark granule was observed in sensitive and resistant *S. marcescens*, due to the action of polymyxin B on the polysaccharide part of the LPS components in the outer membrane [23].

To locate the polysaccharide components in *S. marcescens* cells treated with polymyxin B, the silver proteinate method was used [24]. Blebs are usually formed on the cell surface, due to the aggregation of polymyxin B with LPS and/or phospholipids from the outer membrane of GNB. These blebs contain lipopolysaccharide components, which demonstrates that polymyxin B releases polysaccharide components in the form of lipopolysaccharide [25].

Studies to overcome colistin resistance showed that EDTA may act directly on *P. aeruginosa* to increase its sensitivity to antibiotics and has synergistic potential with colistin or polymyxin B in the treatment of *Pseudomonas* skin or urinary tract infections [26]. EDTA induces an increase in the permeability of the outer membrane in a broad range of GNB. Its mode of action relies on its function as a divalent cation chelator, whereas polymyxin can displace divalent cations by competition [27]. EDTA-resistant mutants release up to 30% of their LPS without dramatic changes in permeability. The released LPS is then split into two parts, one containing the proteins and lipids associated with the LPS, the other containing sugars different from those of the LPS, the latter being responsible for the outer membrane’s permeability. According to this proposal, polymyxin B and EDTA cause similar permeabilization events [27]. The efflux pump inhibitor, carbonyl cyanide m-chlorophenyl hydrazone (CCCP), has also recently demonstrated its efficacy in re-establishing colistin sensitivity in GNB having various resistance mechanisms [28].

### 3.2. Enzymatic Inactivation

Studies carried out several decades ago identified an enzyme capable of inactivating colistin. This enzyme, called colistinase, is a putative serine protease produced by *P. polymyxa*. Briefly, crude colistinase was fractionated into two components (colistinase I and II) by Sephadex G-50 gel filtration. After purification, it was characterized as a single band by polyacrylamide disc gel electrophoresis [29], and it appears to degrade colistin by cleavage of the peptide bond between the tripeptide side chain and the heptapeptide ring [29]. As *P. polymyxa* is a Gram-positive bacterium and therefore lacks LPS, why it develops an apparently secreted enzyme that inactivates colistin remains an intriguing question. Therefore, colistinase may be necessary for survival in the presence of polymyxin [30]. To date, colistin degradation has not been related to colistin resistance in other organisms [30]. Recently, a polymyxin inhibitory enzyme of *Bacillus licheniformis* strain DC-1 has been identified [31]. It is an alkaline Apr protease, which cleaves colistin at two peptide bonds: one between the tripeptide side chain and the heptapeptide ring, and the other between l-Thr and l-Dab within the heptapeptide ring [31]. One of the main contributors to development of antibiotic resistance in bacteria is the acetylation of antibiotics. Recently, Czub et al. described *P. aeruginosa* Gcn5-related N-acetyltransferases (GNAT) which acetylate polymyxin B and polymyxin E. The acetylation process occurs on a single diaminobutyric acid closest to the cyclic peptide [32]. This GNAT appears to be the first enzymatic acylation mechanism with ability to modify polymyxin. Consequently, some recent work has been carried out on GNAT inhibitors [33]. However, it is not known if such enzymatic modification may confer resistance to colistin.

### 3.3. Regulatory System Discovery

The first evidence of *pmrA* (polymyxin resistance A) mutants dates back 42 years, following the analysis of *Salmonella* polymyxin-resistant in vitro mutants [34]. The mutant gene was transferred by conjugation in a colistin-susceptible *S. typhimurium* rough (rfaJ), confirming the role of the mutation in colistin resistance [34]. At that time, research for an altered membrane component in the *pmrA* mutants had so far only given negative results [35]. Vaara et al. showed that polymyxin-resistant *pmrA* strains bind only about 25% of polymyxin compared to the polymyxin-sensitive strain and caused an alteration of the LPS, probably in the lipid A or deep core [35]. Such substitutions reduce the negative charge of the LPS, thus decreasing the accessibility of the positively charged polymyxin to lipid A [36]. Cloning and characterization of a *pmrA* mutant revealed a single base alteration that led to a change in the amino acid in *pmrA* [36]. The mutants were also more resistant to membrane-damaging effects of other cationic agents and EDTA [37]. In 1993, the PmrAB two-component system was characterized from a polymyxin B-resistant mutant strain [38]. An R81H mutation in the PmrA regulator induced an activation constitutive of PmrAB regulation and increased levels of polymyxin resistance [38]. Genetic mapping and DNA sequence analysis subsequently revealed that PmrA is encoded in the *pmrCAB* operon [38]. PmrC, also called *pagB* (*phoP*-activated gene), appears to be a protein involved in LPS modification, while the response regulator PmrA and the kinase sensor *pmrB* form a TCS [39]. The PmrC protein, later known as *EptA*, was described as a phosphoethanolamine transferase acting on lipid A [40].

Microarray, mutagenesis and in silico analysis have demonstrated that the PmrAB system regulates more than 20 confirmed genes, up to 100 genes in *Salmonella*, and orthologs also occur in several Gram-negative pathogens [39]. This TCS seems to be involved predominantly in modifying the cell surface by the addition of Ara4N and pEtN to the LPS. However, it is still somewhat unusual in many ways; it reacts directly to uncommon environmental conditions (high Fe^3+^ content) and is activated indirectly by PhoPQ via the PmrD protein [39], resulting in LPS covalent modifications [36].

**Figure 2:**
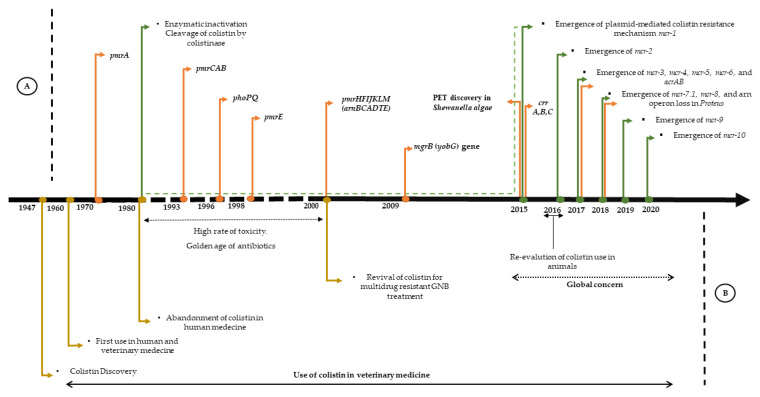
Timeline of the main events related to colistin, from the discovery of colistin to the development of resistance (**A**) represents the described mechanisms, the green arrows symbolize the enzymatic mechanisms, the orange arrows symbolize the chromosomal mechanisms. (**B**) represents the chronology of colistin.

The first report describing the PhoP/PhoQ TCS reported that this system regulates expression of several genes involved in the virulence and survival of *S. typhimurium* in macrophages and confers enhanced susceptibility to the action of cationic antimicrobial peptides (CAMP), called *pag* genes (polymyxin activated genes) [41]. This system has been identified as being a mutation affecting *Salmonella* resistance to raw neutrophil granule extracts, subsequently characterized as a TCS in 1989 [42] (Figure 2). It was believed that the function of this system was to control the expression of a non-specific acidophosphatase [42], based on sequence similarity with homologues of the sensor family. It has been suggested that the protein PhoQ acts as a protein kinase associated with the membrane phosphorylating PhoP in response to environmental signals, in turn activating promoters for *pag* genes. The Pho in PhoP typically refers to loci involved in the metabolism of phosphate [43]. This system reacts to limited concentrations of Mg^2+^ and other divalent cations to activate virulence, and to polymyxin B by interfering with the transcription of more than 40 genes in *Salmonella* [44]. The latter has been identified using either classical genetic methods, Matrix-Assisted Laser Desorption/Ionization—Time of Flight (MALDI-TOF) analysis, or high-density DNA chips [43]. Indeed, transposon mutagenesis has shown that this system represents a regulator, and that it was necessary for the expression of a panel of genes involved in virulence with different chromosomal locations, such as *pagC* [41]. In an *E. coli* K-12 and upstream of the transcription initiation site of three PhoP-activated genes, namely *phoPQ*, *mgtA,* and *mgrB* at 25 bp, a direct repetition, (T / G) GTTTA, has been identified, representing a PhoP liaison site [43]. This pattern has also been identified in the promoter region of the *pmrA* gene [43]. The analysis of these various genes regulated by PhoP leads to the following conclusions: this PhoP/PhoQ regulator is involved in the adaptation to Mg^2+^ restrictive environments, it regulates virulence in several bacterial species; several genes regulated by PhoP are species-specific, and this system regulates the modification of many components of the bacterial cell envelope [43]. The TCS PhoP/PhoQ regulates LPS changes by the addition of aminoarabinose and 2-hydroxy myristate through the extraction of lipid A and LPS from different strains of *S. typhimurium* [45]. The fatty acid content of LPS and whole bacteria has been investigated by gas chromatography (GC) mass spectrometry [45].

### 3.4. Arn Operon Discovery

Gunn et al. reported the identification of two PmrA/PmrB regulated loci necessary for the addition of aminoarabinose to lipid A and thus for polymyxin resistance in *S. typhimurium* [46]. Analysis of the DNA sequence extending the transposon showed that the insertions were located in *pagA* (now called *pmrE*), a gene regulated by PhoP/PhoQ and activated by the previously identified PmrA/PmrB, which is a UDP-glucose dehydrogenase [46]. Mass spectrometry of the mutant *pmrE* showed that it is impossible to add aminoarabinose to the LPS, and that it is transcribed as an individual unit [46]. For insertion into the second gene, it appears to be driven by a seven-gene operon, named *pmrHFIJKLM* [47], an active component in naturally colistin-resistant GNB [48] (Figure 2).

### 3.5. mgrB (yobG) Discovery

The *mgrB* was first described in 1999 in *E. coli* as a short lipoprotein, induced by low extracellular levels of magnesium and controlled by the PhoP/PhoQ two-component system [49]. It was further demonstrated that the *phoP* protein binds in vitro to the *mgrB* promoter region responding to Mg^2+^ [49]. The *mgrB* gene, also known as *yobG*, identified in *Salmonella enterica*, has been shown to be activated by PhoP through microarray experiments and transcription reports [50] (Figure 2). The *mgrB* gene was discovered to be a regulator of the PhoP/PhoQ pathway, based on the hypothesis of feedback inhibition in terms of stress response. The researchers screened *phoP*-regulated genes individually in *E. coli* by knockout [50]. The deletion of *mgrB* coding for a small membrane protein of only 47 amino acids led to upregulation of the PhoP/PhoQ regulator system in *E. coli*, *S. typhimurium*, *Yersinia pestis,* and other related bacteria [50]. The sequence alignments showed multiple conserved residues, suggesting a well-conserved negative regulatory system [50]. The proper annotation of genes encoding for small proteins such as the *mgrB* gene remains a major challenge. The open reading frame of the *mgrB* gene was predicted based on the existence of a ribosomal binding site within an intergenic region [51]. Inactivation of *mgrB* through intergenic sequences represents one of the most common colistin resistance mechanisms and is regarded as an emerging epidemic [52,53].

### 3.6. CrrAB Discovery

Investigation of colistin resistance mechanisms in nine *K. pneumoniae* clinical isolates, through a combination of genome sequencing and RNA sequencing transcriptional profile analysis (RNA-Seq) [54], illustrated a range of genetic variations. This enabled the characterization of distinct mutations within two strains in a previously uncharacterized histidine kinase gene belonging to a TCS named CrrAB (Figure 2), which was found to be highly expressed, as well as *pmrCAB* and an adjacent glycosyltransferase gene [54]. Complementation assays with the wild-type gene restored colistin sensitivity in both strains. The *crrAB* genes are common in most *K. pneumoniae* genomes and the resistance mechanisms are genetically background-dependent [54].

## 4. Exploitation of Colistin Resistance: Methods and Challenges

### 4.1. Lipid A Extraction and Mass Spectrometry

Historically, mass spectrometry has been used in colistin resistance to highlight lipid A modifications in colistin-resistant bacteria. MALDI-TOF MS directly assesses the biochemical cause of colistin resistance and the presence, absence and modification of lipid A, the capsule and the LPS structure, which are components of colistin resistance [9], as lipid A has a specific peak of 1796.2 m/z [55]. Chromosomal mutations cause mass shifts of m/z +131, +123, and/or +161 in lipid A through the substitution of at least one phosphate with 4-amino-4-deoxy-L-arabinose (L-Ara4N), phosphoethanolamine (pEtN) and/or galactosamine, respectively [56]. As for the *mcr-1* gene, a displacement of m/z+123 has been observed in the lipid A peaks of Gram-negative colistin-resistant pathogens [56].

The development of MALDI-TOF MS technology, combined with automation and the implementation of a user-friendly interface in clinical laboratories, has considerably changed bacteriological diagnoses and opened the possibility of developing new rapid tools for the detection of colistin resistance. First, MALDI-TOF ionization for clinical microbiology laboratories was developed in positive ion mode, and despite numerous studies devoted to the mechanistic understanding of the ionization principle, few studies have been devoted to analysis in negative ion mode [57]. Recently, some new machines used the negative mode, which makes it possible to analyse lipids and therefore lipid A [57,58]. The analytical method requires a more elaborate sample preparation than that applied for routine MALDI-TOF identification, involving hydrolysis of LPS and extraction of lipid A by solvents [56]. This method has recently been simplified and made chemically safer, done in 30 min [59].

Thus, three studies evaluated MALDIxin, a test based on new MALDI-TOF machines [60,61,62]. The MALDIxin assay was developed to detect pEtN modification in lipid A directly from bacterial colonies in less than 15 min. Dortet et al. (2018) evaluated MALDIxin on *Acinetobacter baumannii* isolates. Peaks m/z 2033.3 and m/z 1935.3 were associated with pEtN-modified lipid A in all colistin-resistant isolates and were not observed in any of the colistin-sensitive isolates [60]. The optimization of MALDIxin allowed identification of L-Ara4N and pEtN-modified lipid A in *E. coli* and *K. pneumoniae* isolates. In addition, the optimized methods were able to discriminate chromosomally encoded colistin resistance and *mcr*-mediated colistin resistance, by comparing the percentage of modification of Lipid A by either L-Ara4N or pEtN [61,62]. For chromosomal resistance, a 100% of modification through the addition of L-Ara4N was observed, while for *mcr*-encoded resistance, up to 100% of the modified Lipid A was related to the addition of pEtN [62].

### 4.2. The Discovery of New Variants and Mutations in a Common Gene

The PCR-based approach (conventional PCR, real-time qRT-PCR) is the most common molecular method used by clinical laboratories to understand resistance mechanisms underlying the observed phenotypic resistance to colistin [9]. Such approaches are used to amplify the known genes involved in colistin resistance: *mgrB*, *phoPQ*, *pmrAB*, *pmrHFIJKLM/arnBCADT*, and *mcr* genes. The amplicons are sequenced and analysed by comparing the sequences to wild-type strain sequences for possible mutation of the amplified gene. In addition, qRT-PCR is also used to study the expression levels of these genes in both strains to determine the transcription rate of the respective resistance genes in the resistant phenotype [9].

### 4.3. Genetic Toolbox and Colistin Resistance Research

Functional genomics: enables the identification of new antibiotic resistance genes encoding for an enzyme based on their function in an expression vector. However, it becomes meaningless if the mechanisms of resistance involve several components or regulatory genes [63,64]. Functional genomics is based on the extraction and fragmentation of DNA enabling the creation of a specific library of a homogeneous size. This library is then cloned into an expression vector, which is then screened depending on the studied antibiotic [64]. This strategy has been used to decipher the colistin resistance mechanism in *Shewanella algae* MARS 14, suggesting the involvement of a phosphoethanolamine transferase *EptA* coding for *pmrC* in polymyxin resistance [63].

Mutagenesis: variations in microorganism genomes are often the result of homologous recombination that allows the exchange of genes without reorganizing the genome. Other sources of variations come from the environment, such as plasmids or from linear DNA sequences that can move through the genome (transposon) [65]. Transposon-based mutagenesis of bacterial genomes is a powerful method for indirectly identifying genetic elements associated with specific functions and phenotypes by the creation of mutations at random locations in genomes [66]. Tn5 and Tn10 transposases, both used to generate non-redundant mutant libraries, are among the most frequently used transposon tools [67,68,69].

Random mutagenesis methods have been widely used since the 1980s to elucidate the main genes involved in colistin resistance, as described above. At the time, these methods were complex, due to the difficulty of sequencing the genes of interest and analysing the proteins involved. Thus, colistin resistance mechanisms have been studied mainly in *E. coli* and *S. enterica* [3]. These techniques are now used to identify the molecular mechanisms of colistin resistance in other species. The transposon is randomly inserted into the bacterial chromosome, generating a random library of mutants which can be screened by modifying the culture medium, depending on the studied phenotype [64]. Recently, transposon mutagenesis has highlighted the role of certain efflux pumps in colistin resistance mechanisms [64]. Telke et al. demonstrated that heteroresistance is linked to overexpression of the *acrAB-tolC* efflux pump in *Enterobacter spp*. after the screening of mutant clones generated by transposon mutagenesis [70]. More recently, Huang et al. showed that the *phoP* gene, the *dedA*(*Ecl*) gene (coding for an inner membrane protein of the *DedA* family) and the *tolC* gene (coding for part of the *AcrAB-TolC* efflux pump) are required for colistin heteroresistance, and they identified a new gene, *ecr*, coding for a transmembrane protein of 72 amino acids, which mediates colistin heteroresistance in *Enterobacter cloacae* [71].

The Himar1 mariner is a novel transposon isolated from the barn fly *Haematobia irritans*, identified by Lampe et al. and has become popular in bacterial genetics [72,73]. This system encodes a transposase and a transposon within one or two suicide plasmids that are electroporated on host cell-free bacteria. Transposase Himar1 randomly integrates the transposon, which contains an antibiotic resistance cassette and fluorescent markers that are flanked by repeats into the AT dinucleotide sites of the bacterial genome through a cut and paste process, which allows dual selection. The himar1 system has so far remained unexploited in the field of colistin resistance, despite its advantage of genetic markers in the transposon which facilitate selection, visual detection, and identification of insertion loci in the bacterial genome [65].

### 4.4. Whole Genome Sequencing: A Modern Approach for a Growing Challenge

The development of genomic techniques allowing the study of diverse environmental microbial communities independently of culture has been a key factor in the paradigm changes in resistance studies [74]. Modern high-throughput DNA sequencing technologies have the potential to contain the growing antibiotic resistance trend. Analysis of the genome sequences of large collections of strains has elucidated the pathways by which antibiotic-resistant bacteria propagate [75]. Furthermore, metagenomic sequencing has revealed a large reservoir of antibiotic resistance genes present in the microbial community complex that populate the gastrointestinal tract of animals, humans, and natural environments such as surface water or soil [76]. This is demonstrated by the growing number of known resistance genes, which has occurred in parallel with the rapid decrease in sequencing costs [74]. Genomic analysis combined with updated databases is an emerging alternative approach that provides access to large amounts of information and has helped in studying resistance and better elucidating bacterial behaviour [55].

In late 2015, a Chinese team for the first time demonstrated the emergence of a colistin resistance gene carried by a plasmid, the *mcr-1* gene [5] (Figure 2). The existence of this plasmid-mediated colistin-resistance gene was demonstrated by conjugation. The *mcr-1* gene was then identified by WGS and homology modelling of the extracted plasmid pHNSHP45, using an IncI2 plasmid (pHN1122-1) as reference for annotation [5]. The *mcr-1* gene was ligated to a pUC18 cloning vector and transformed into *E. coli*. This transformant had a 4-fold increase in MIC, indicating that *mcr-1* resistance was conferred for colistin. The researchers undertook conjugation and transformation experiments to confirm the transferability of this gene [5]. Using an amino acid homology sequence, they identified *mcr-1* codes for a plasmid phosphoethanolamine transferase that catalyses the addition of pEtN to lipid A, increasing the cationic charges on LPS, thus decreasing the affinity of colistin for LPS [5]. Through genetic and bioinformatic analyses, the origin of the gene could be linked to the genus *Moraxella*. The *mcr-1* gene appears to be carried by various types of plasmids (IncX4, IncI2, IncHI2, IncF, IncY, and IncP) [7]. Since then, *mcr-1* has been reported worldwide in over 40 countries in 5 continents, and among 11 *Enterobacteriaceae* species [77]. International genome databases (NCBI, EBI) have facilitated access to genomic data, studying the source and identifying new variants of this *mcr* gene. Thus, recent publications have suggested different groups of origin: *Moraxella*, *Enhydrobacter*, *Methylophilaceae*, *Limnobacter,* and *Vibrio* as primary sources of the gene [78]. Most of the described mcr gene variants originate from animals or humans and bacteria present in water sources, which may suggest defensive functions against bacteriophages or antimicrobial peptides [78]. The pathway by which the *mcr-1* gene circulates and spreads has remained ambiguous [7,77]. The use of colistin in veterinary medicine has probably accelerated the worldwide dissemination of the *mcr-1* gene among animals and humans, which is consistent with the hypothesis that cattle, and particularly pigs, are most likely the primary source of *mcr-1* producers [7,77]. After the prohibition of colistin use as a growth promoter in animals, a considerable reduction in colistin resistance in animals and humans has been observed [79].

*mcr-2* has been discovered in an *E. coli* strain isolated from pigs and cattle in Belgium. Exclusively carried by a type IncX4 plasmid, the progenitor of the gene coding for *mcr-2* is most likely *Moraxella pluranimalium* [80] *mcr-3*, carried by an IncHI2 type plasmid. A bacterial species of the genus *Aeromonas* would be the progenitor of the *mcr-3* [81]. *mcr-4*, carried by a colE10 plasmid, was discovered in a strain of *S. enterica* serovar *Typhimurium* isolated from pigs in Italy and in *E. coli* strains from pigs in Spain and Belgium and may have *Shewanella frigidimarina* as progenitor [82]. The *mcr-5* gene was detected in an *S. enterica* serovar *Paratyphi B* carried by a ColE plasmid [83]. Until now, the progenitor is unidentified, but the *mcr-5* protein was identified in *Cupriavidus gilardii*, suggesting that this bacterial species could be the progenitor [7]. The variant *mcr-2.2* has been discovered in *Moraxella spp*. and renamed *mcr-6* [7]. One variant has been identified. The *mcr-7* gene has been identified in *K. pneumoniae* hosted on an IncI2 type conjugated plasmid. Like *mcr-3*, *mcr-7.1* comes from the *Aeromonas* species. One variant of *mcr-7* (*mcr-7.1*) has been identified to date [84]. *mcr-8* is carried on an IncFII-type conjugative plasmid in *K. pneumoniae*. Four variants of *mcr-8* have been identified, *mcr-8.1* to *mcr-8.4* [85]. *mcr-9* was discovered during an in silico screening of antimicrobial resistance gene of *S. typhimurium* sequenced genomes; the gene was harboured by IncHI2 plasmids [86]. *mcr-10* has been identified in an *Enterobacter roggenkampii* strain carried by an IncFIA plasmid. It seems the proteins *mcr-10* and *mcr-9* derived from a common ancestor of the *Buttiauxella* genes [87]. Generally, only one *mcr* determinant is contained in a bacterial isolate. However, recent studies have demonstrated the co-existence of different *mcr* variants in the same bacterial strain [88].

The analysis of 64,628 genomes deposited in NCBI databases and screened for *mcr* sequences predicted a total of 5265 putative *mcr*-like sequences that may soon be described in pathogenic bacteria, as was done for all the previously mentioned mcr variants. The study of the genetic environment demonstrated that the three less commonly found genes lack pap2- or dgkA-linked sequences, like *mcr-4* and *mcr-5*, or are clustered to a truncated pap2 coding sequence in the even more rarely found *mcr-2* gene [89]. Furthermore, *mcr-9* and *mcr-10*, the two last and most closely related *mcr* genes identified so far, lack both hpap2 and dgkA sequences [89].

### 4.5. Transcriptomics and Proteomics

Barczak and colleagues suggest that when using genome sequencing, the prediction of antimicrobial resistance requires a prior knowledge of all the variables that can induce phenotypical resistance (inactivating enzymes, porin mutations, influx systems, binding site mutations, gene inactivation, promoter mutations, etc.) [90]. They underline that sequence-based known resistance markers represent a small proportion and believe that transcriptome analysis could possibly afford a more comprehensive phenotypic profile [90]. They also showed that changes in bacterial expression patterns in the presence or absence of antibiotics may differ, offering the possibility of detecting resistance at the RNA level as a function of stress responses. The study of RNA provides a viable alternative to the research for resistance genes in genome sequences [91]. High throughput RNA sequencing is a modern “omics” technology [91], which employs deep sequencing technologies and is based on the sequencing of an RNA population converted into a library of cDNA fragments [92]. The read sequences are then assembled bioinformatically to reconstruct the complete transcription sequence and quantify the expression levels of the annotated genes [92].

The study of transcriptome profile variation has emerged as a widely used method to explore the genetic mechanisms that confer colistin resistance. Using a combination of genome sequencing and transcriptional profiling by RNA sequencing analysis (RNA-Seq), the identification of *crr* genes previously described as an uncharacterized histidine kinase as additional regulators of colistin resistance broadens the available genes that regulate the phenotype and illustrates the multiplicity of ways in which bacteria respond to antimicrobial peptides [54]. The increased expression of cation transporters and other efflux pumps are among the transcription modifications commonly found in colistin-resistant strains [54].

Proteome profiling has always started with immobilized pH gradient two-dimensional gel electrophoresis (2-DE) to identify differential protein abundance between samples, with validation by qPCR and/or western blot [93]. Proteomics and metabolomics, other new “omics” approaches, are used to elucidate the development process of specific biologicals and regulatory mechanisms, as well as the expression of the entire protein and changes in metabolites in specific tissues or cells at the system level [94]. The use of these “omics” techniques has enabled the large-scale discovery of new potential targets to investigate colistin resistance [95]. Fernández-Reyes et al. studied the cost of colistin-resistant *A. baumannii* resistance. They revealed that 35 differently expressed proteins, including phosphorodiamidate morpholino oligomers (PMOs), chaperones, enzymes involved in metabolism, and protein biosynthesis factors, were down-regulated in colistin-resistant strains [96]. An unlabelled quantitative proteomics study compared the proteomes of *mcr-1*-mediated colistin-resistant and colistin-sensitive *E. coli*. The authors identified a large amount of differentially expressed proteins that may contribute to *mcr-1*-mediated antimicrobial resistance through regulation of glycerophospholipid metabolism, LPS biosynthesis and phosphoethanolamine substrate accumulation [94]. Additionally, to adapt to colistin stress, some proteins involved in the TCA cycle and pentose phosphate pathway were highly expressed in *K. pneumoniae* strains [97]. These recent advances in proteomics highlight significant metabolic pathways and suggest that the processes involved in the acquisition of the colistin response are related to energy, carbohydrate, and lipid metabolism [96,97]. Thus, the inhibition of the metabolic process may potentially offer a way to overcome colistin resistance [97].

Proteomics offers a more profound, global perspective on the molecular mechanisms of polymyxin resistance and should be applied in conjunction with other omics approaches: genomics, transcriptomics, and metabolomics, in order to understand polymyxin resistance [93]. It is still widely unexplored, due to the complexity of the strains, the cost of running proteomics on a large number of samples, the amount of generated data, and the difficulty of interpreting results [93].

### 4.6. Application of CRISPR-cas 9-Based Genome Editing

Over the past 10 years, clustered regularly interspaced short palindromic repeats, the CRISPR-Cas Type II system of Streptococcus pyogenes, have become the easier, faster and predominant choice for engineering applications of the genomes [98]. This system requires the co-expression of the Cas9 protein and two RNAs that guide Cas9 to the target site in the intrusive DNA for recognition and subsequent cleavage. Genome editing with Cas9 has become easier since both RNAs have been enhanced to become a chimeric single-guided RNA (sgRNA). The gRNA targeting specific sequences recruits the Cas9 protein to form the compound, and the Cas9 protein acts as a nuclease and generates a blunt end double-strand break [99].

So far, the CRISPR/Cas9 system has been used as a new microbial gene therapy technology to combat colistin resistance, to confirm its use among resistant bacteria [100] and to investigate determinants of antimicrobial resistance. However, this technique is limited to in vitro treatment, due to its inability to penetrate the cell membrane by itself. There is also the lack of an effective delivery system for CRISPR/Cas9 systems, limited to phage based CRISPR/Cas9 dissemination strategies, with a risk of recombination of drug resistance genes, a narrow host range, diffusion barriers and bacterial resistance to phages [100,101]. Recently, bacterial conjugation seems to be one of the ways to release CRISPR/Cas9 into bacteria through the construction of a host-independent conjugated plasmid, which has effectively and selectively eliminated antibiotic resistance genes in *E. faecalis* [102]. Some recent original work provides a convincing demonstration of the potential of CRISPR/Cas technology to overcome the problem of multi-drug resistance, by demonstrating that existing bacterial CRISPR/Cas systems can limit the spread of drug resistance genes by countering multiple horizontal gene transfer pathways [103]. This can limit the spread of plasmid resistance by targeting antibiotic resistance genes and destroying bacterial plasmids conferring resistance [104,105]. Combating antibiotic-resistant bacteria by eliminating resistance genes contained in a bacterial community without affecting it has become possible with the advent of CRISPR/Cas9, through anti-plasmid approaches [106]. Recently, the curing of specific plasmids in multiple plasmid-carrying bacteria through the Cas9 gRNA system has been developed [106]. This system has demonstrated high efficiency, and it has been effective in destroying the plasmid harbouring *mcr-1* in *E. coli* [100] and in overcoming the resistance to other antibiotics, such as the carbapenemase and extended-spectrum-lactamase (ESBL) eradication in clinical isolates of Enterobacteriaceae [106,107]. Sun et al. have developed the CRISPR/Cas9 editing system to delete the *ramR*, *tetA* for tigecycline, and the *mgrB* gene for colistin [108] and to study the effect of these genes in carbapenem-resistant *K. pneumoniae*. More recently, McConville et al. built a CRISPR system to insert a single nucleotide point (SNP) mutation in *crrB*, *arnT* and to disrupt *crrA*, *crrB*, *mgrB,* and *pmrA* genes. *crrB* gene mutations lead to resistance to polymyxins and mediate the addition of both L-Ara4N and pEtN to lipid A, thus efficiently validating the functions of these genes [109]. This technology has the advantage of incorporating the desired mutations that enable a specific and precise modification at a particular site of the bacterial genome, without affecting the rest of the genome [110].

## 5. Conclusions

Due to its disuse in clinical medicine for years, the mechanisms of action and resistance to colistin remained unclear for a long time. This delay is compounded by a complex mechanism involving multiple metabolic pathways. However, the scientific impetus of recent years has enabled us to increase our knowledge in this field, supported by the use and development of innovative tools. For example, whole genome sequencing represents a real added value to more traditional in vitro methods. It also enables faster detection of the presence of mutations in genes known to be involved in colistin resistance, although in vitro confirmation is still required. High throughput transcriptomics tools have considerably increased the data pool on colistin resistance. However, they remain expensive, difficult to interpret and isolate-dependent. Only a few such studies have been conducted, and the accumulation of data, combined with genome sequencing data, may allow us to identify the pathways involved in resistance. Finally, new knock-in/knock-out strategies and mutagenesis methods represent powerful tools for research in this field. Modern biotechnology techniques, high-throughput sequencing, omics techniques, and genome manipulation have facilitated a new era of discovery. Despite spectacular progress, challenges to the detection and understanding of antimicrobial resistance persist, especially for colistin, due to the unexpectedly large number of mechanisms described in various bacterial species.

## Data Availability

Not applicable.

## References

[1] Baron S., Hadjadj L., Rolain J.M., Olaitan A.O. (2016). Molecular mechanisms of polymyxin resistance: Knowns and unknowns. Int. J. Antimicrob. Agents.

[2] Olaitan A.O., Morand S., Rolain J.M. (2014). Mechanisms of polymyxin resistance: Acquired and intrinsic resistance in bacteria. Front. Microbiol..

[3] Poirel L., Jayol A., Nordmann P. (2017). Polymyxins: Antibacterial activity, susceptibility testing, and resistance mechanisms encoded by plasmids or chromosomes. Clin. Microbiol. Rev..

[4] Texte I.P.A. (1961). du Annales de l’Institut Pasteur: Journal de Microbiologie/Publiées sous le Patronage de M. Pasteur par E. Duclaux. https://gallica.bnf.fr/ark:/12148/cb34348753q/date.

[5] Liu Y.Y., Wang Y., Walsh T.R., Yi L.X., Zhang R., Spencer J., Doi Y., Tian G., Dong B., Huang X. (2016). Emergence of plasmid-mediated colistin resistance mechanism MCR-1 in animals and human beings in China: A microbiological and molecular biological study. Lancet Infect. Dis..

[6] Stansly P.G., Schlosser M.E. (1947). Studies on Polymyxin: Isolation and Identification of Bacillus polymyxa and Differentiation of Polymyxin from Certain Known Antibiotics. J. Bacteriol..

[7] Ahmed E.S.M.A.E.G., Zhong L.L., Shen C., Yang Y., Doi Y., Tian G.B. (2020). Colistin and its role in the Era of antibiotic resistance: An extended review (2000–2019). Emerg. Microbes Infect..

[8] Kaye K.S., Pogue J.M., Tran T.B., Nation R.L., Li J. (2016). Agents of Last Resort: Polymyxin Resistance. Infect. Dis. Clin. North Am..

[9] Sekyere O.J., Govinden U., Bester L.A., Essack S.Y. (2016). Colistin and tigecycline resistance in carbapenemase-producing Gram-negative bacteria: Emerging resistance mechanisms and detection methods. J. Appl. Microbiol..

[10] Haenni M., Poirel L., Kieffer N., Châtre P., Saras E., Métayer V., Dumoulin R., Nordmann P., Madec J.Y. (2016). Co-occurrence of extended spectrum lactamase and MCR-1 encoding genes on plasmids. Lancet Infect. Dis..

[11] Lopes J., Inniss W.E. (1969). Electron microscopy of effect of polymyxin on Escherichia coli lipopolysaccharide. J. Bacteriol..

[12] Koike M., Iida K., Matsuo T. (1969). Electron microscopic studies on mode of action of polymyxin. J. Bacteriol..

[13] Storm D.R., Rosenthal K.S., Swanson P.E. (1977). Polymyxin and related peptide antibiotics. Annu. Rev. Biochem..

[14] Newton B.A. (1954). Site of action of polymyxin on Pseudomonas aeruginosa: Antagonism by cations. J. Gen. Microbiol..

[15] Schindler P.R.G., Teuber M. (1975). Action of polymyxin B on bacterial membranes: Morphological changes in the cytoplasm and in the outer membrane of Salmonella typhimurium and Escherichia coli B. Antimicrob. Agents Chemother..

[16] Newton B.A. (1956). The properties and mode of action of the polymyxins. Microbiol. Mol. Biol. Rev..

[17] Few A.V. (1955). The interaction of polymyxin E with bacterial and other lipids. BBA-Biochim. Biophys. Acta.

[18] Petersdorf R.G., Plorde J.J. (1963). Colistin-A Reappraisal. JAMA J. Am. Med. Assoc..

[19] Conrad R.S., Wulf R.G., Clay D.L. (1979). Effects of carbon sources of antibiotic resistance in pseudomonas aeruginosa. Antimicrob. Agents Chemother..

[20] Haas G.J., Sevag M.G. (1953). Critical role of amino acids on the sensitivity and development of resistance to polymyxin B. Arch. Biochem. Biophys..

[21] Shimizu S., Iyobe S., Mitsuhashi S. (1977). Inducible high resistance to colistin in Proteus strains. Antimicrob. Agents Chemother..

[22] Sud I.J., Feingold D.S. (1970). Mechanism of polymyxin B resistance in Proteus mirabilis. J. Bacteriol..

[23] Weber D., Nadakavukaren M., Tsang J. (1979). Electron microscopic observations of polysaccharide components in polymyxin b treated outer membranes from serratia marcescens. J. Antibiot. (Tokyo).

[24] Thiery J.P. (1967). Mise en evidence des polysaccharides sur coupes fines en microscopie electronique. J. Microsc..

[25] Weber D.A., Nadakavukaren M.J., Tsang J.C. (1978). Localization of polysaccharide components in polymyxin b treated cells of serra tia marcescens. J. Antibiot. (Tokyo).

[26] Davis S.D., Iannetta A., Wedgwood R.J. (1971). Activity of colistin against pseudomonas aeruginosa: Inhibition by calcium. J. Infect. Dis..

[27] Hancock R.E.W. (1984). Alterations in structure of the cell envelope. Ann. Rev. Microbiol..

[28] Baron S.A., Rolain J.M. (2018). Efflux pump inhibitor CCCP to rescue colistin susceptibility in mcr-1 plasmid-mediated colistin-resistant strains and Gram-negative bacteria. J. Antimicrob. Chemother..

[29] Kagawa I.M., Koyama Y. (1980). Selective cleavage of a peptide antibiotic, colistin by colistinase. J. Antibiot. (Tokyo).

[30] Moffatt J.H., Harper M., Boyce J.D. (2019). Mechanisms of Polymyxin Resistance. Advances in Experimental Medicine and Biology.

[31] Yin J., Wang G., Cheng D., Fu J., Qiu J., Yu Z. (2019). Inactivation of polymyxin by hydrolytic mechanism. Antimicrob. Agents Chemother..

[32] Czub M.P., Zhang B., Chiarelli M.P., Majorek K.A., Joe L., Porebski P.J., Revilla A., Wu W., Becker D.P., Minor W. (2018). A Gcn5-Related N-Acetyltransferase (GNAT) Capable of Acetylating Polymyxin B and Colistin Antibiotics in Vitro. Biochemistry.

[33] Burckhardt R.M., Semerena E.J.C. (2020). Small-Molecule Acetylation by GCN5-Related N -Acetyltransferases in Bacteria. Microbiol. Mol. Biol. Rev..

[34] Mäkelä H.P., Sarvas M., Calcagno S., Lounatmaa K. (1978). Isolation and genetic characterization of polymyxin-resistant mutants of Salmonella. FEMS Microbiol. Lett..

[35] Vaara M., Vaara T., Sarvas M. (1979). Decreased binding of polymyxin by polymyxin-resistant mutants of Salmonella typhimurium. J. Bacteriol..

[36] Gunn J.S., Miller S.I. (1996). PhoP-PhoQ activates transcription of pmrAB, encoding a two-component regulatory system involved in Salmonella typhimurium antimicrobial peptide resistance. J. Bacteriol..

[37] Vaara M. (1981). Increased outer membrane resistance to ethylenediaminetetraacetate and cations in novel lipid A mutants. J. Bacteriol..

[38] Roland K.L., Martin L.E., Esther C.R., Spitznagel J.K. (1993). Spontaneous pmrA mutants of Salmonella typhimurium LT2 define a new two- component regulatory system with a possible role in virulence. J. Bacteriol..

[39] Gunn J.S. (2008). The Salmonella PmrAB regulon: Lipopolysaccharide modifications, antimicrobial peptide resistance and more. Trends Microbiol..

[40] Lee H., Hsu F.F., Turk J., Groisman E.A. (2004). The PmrA-regulated pmrC gene mediates phosphoethanolamine modification of lipid A and polymyxin resistance in Salmonella enterica. J. Bacteriol..

[41] Miller S.I., Kukral A.M., Mekalanos J.J. (1989). A two-component regulatory system (phoP phoQ) controls Salmonella typhimurium virulence. Proc. Natl. Acad. Sci. USA.

[42] Kier L.D., Weppelman R.M., Ames B.N. (1979). Regulation of nonspecific acid phosphatase in Salmonella: phoN and phoP genes. J. Bacteriol..

[43] Groisman E.A. (2001). The pleiotropic two-component regulatory system PhoP-PhoQ. J. Bacteriol..

[44] McPhee J.B., Lewenza S., Hancock R.E.W. (2003). Cationic antimicrobial peptides activate a two-component regulatory system, PmrA-PmrB, that regulates resistance to polymyxin B and cationic antimicrobial peptides in Pseudomonas aeruginosa. Mol. Microbiol..

[45] Guo L., Lim K.B., Gunn J.S., Bainbridge B., Darveau R.P., Hackett M., Miller S.I. (1997). Regulation of lipid A modifications by Salmonella typhimurium virulence genes phoP-phoQ. Science.

[46] Gunn J.S., Lim K.B., Krueger J., Kim K., Guo L., Hackett M., Miller S.I. (1998). PmrA-PmrB-regulated genes necessary for 4-aminoarabinose lipid A modification and polymyxin resistance. Mol. Microbiol..

[47] Gunn J.S., Ryan S.S., Van Velkinburgh J.C., Ernst R.K., Miller S.I. (2000). Genetic and functional analysis of a PmrA-PmrB-regulated locus necessary for lipopolysaccharide modification, antimicrobial peptide resistance, and oral virulence of Salmonella enterica serovar typhimurium. Infect. Immun..

[48] Baron S., Leulmi Z., Villard C., Olaitan A.O., Telke A.A., Rolain J.M. (2018). Inactivation of the arn operon and loss of aminoarabinose on lipopolysaccharide as the cause of susceptibility to colistin in an atypical clinical isolate of proteus vulgaris. Int. J. Antimicrob. Agents.

[49] Kato A., Tanabe H., Utsumi R. (1999). Molecular characterization of the PhoP-PhoQ two-component system in Escherichia coli K-12: Identification of extracellular Mg2+-responsive promoters. J. Bacteriol..

[50] Lippa A.M., Goulian M. (2009). Feedback inhibition in the PhoQ/PhoP signaling system by a membrane peptide. PLoS Genet..

[51] Hemm M.R., Paul B.J., Schneider T.D., Storz G., Rudd K.E. (2008). Small membrane proteins found by comparative genomics and ribosome binding site models. Mol. Microbiol..

[52] Mouna H., Stylianos C., Linda H., Efthimia P., Sophia P., Nikoletta C., Sophia T., Vassiliki P., Nikoletta S., Iris S. (2020). Inactivation of mgrB gene regulator and resistance to colistin is becoming endemic in carbapenem-resistant Klebsiella pneumoniae in Greece: A nationwide study from 2014 to 2017. Int. J. Antimicrob. Agents.

[53] Olaitan A.O., Diene S.M., Kempf M., Berrazeg M., Bakour S., Gupta S.K., Thongmalayvong B., Akkhavong K., Somphavong S., Paboriboune P. (2014). Worldwide emergence of colistin resistance in Klebsiella pneumoniae from healthy humans and patients in Lao PDR, Thailand, Israel, Nigeria and France owing to inactivation of the PhoP/PhoQ regulator mgrB: An epidemiological and molecular study. Int. J. Antimicrob. Agents.

[54] Wright M.S., Suzuki Y., Jones M.B., Marshall S.H., Rudin S.D., Van Duin D., Kaye K., Jacobs M.R., Bonomo R.A., Adamsa M.D. (2015). Genomic and transcriptomic analyses of colistin-resistant clinical isolates of Klebsiella pneumoniae reveal multiple pathways of resistance. Antimicrob. Agents Chemother..

[55] Bardet L., Rolain J.M. (2018). Development of new tools to detect colistin-resistance among enterobacteriaceae strains. Can. J. Infect. Dis. Med. Microbiol..

[56] Welker M., Van Belkum A., Girard V., Charrier J.P., Pincus D. (2019). An update on the routine application of MALDI-TOF MS in clinical microbiology. Expert Rev. Proteom..

[57] Leopold J., Popkova Y., Engel K., Schiller J. (2018). Recent Developments of Useful MALDI Matrices for the Mass Spectrometric Characterization of Lipids. Biomolecules.

[58] Amano J., Sugahara D., Osumi K., Tanaka K.K. (2009). Negative-ion MALDI-QIT-TOFMSn for structural determination of fucosylated and sialylated oligosaccharides labeled with a pyrene derivative. Glycobiology.

[59] Maumus L.G., Clements A., Filloux A., McCarthy R.R., Mostowy S. (2016). Direct detection of lipid A on intact Gram-negative bacteria by MALDI-TOF mass spectrometry. J. Microbiol. Methods.

[60] Dortet L., Potron A., Bonnin R.A., Plesiat P., Naas T., Filloux A., Maumus L.G. (2018). Rapid detection of colistin resistance in Acinetobacter baumannii using MALDI-TOF-based lipidomics on intact bacteria. Sci. Rep..

[61] Furniss R.C.D., Dortet L., Bolland W., Drews O., Sparbier K., Bonnin R.A., Filloux A., Kostrzewa M., Mavridou D.A.I., Maumus L.G. (2019). Detection of colistin resistance in Escherichia coli by use of the MALDI biotyper sirius mass spectrometry system. J. Clin. Microbiol..

[62] Dortet L., Broda A., Bernabeu S., Glupczynski Y., Bogaerts P., Bonnin R., Naas T., Filloux A., Maumus L.G. (2020). Optimization of the MALDIxin test for the rapid identification of colistin resistance in Klebsiella pneumoniae using MALDI-TOF MS. J. Antimicrob. Chemother..

[63] Telke A.A., Rolain J.M. (2015). Functional genomics to discover antibiotic resistance genes: The paradigm of resistance to colistin mediated by ethanolamine phosphotransferase in Shewanella algae MARS 14. Int. J. Antimicrob. Agents.

[64] Hadjadj L., Baron S.A., Diene S.M., Rolain J.M. (2019). How to discover new antibiotic resistance genes?. Expert Rev. Mol. Diagn..

[65] McClure E.E., Chávez A.S.O., Shaw D.K., Carlyon J.A., Ganta R.R., Noh S.M., Wood D.O., Bavoil P.M., Brayton K.A., Martinez J.J. (2017). Engineering of obligate intracellular bacteria: Progress, challenges and paradigms. Nat. Rev. Microbiol..

[66] Kulasekara H.D. (2014). Transposon mutagenesis. Methods Mol. Biol..

[67] Way J.C., Davis M.A., Morisato D., Roberts D.E., Kleckner N. (1984). New Tn10 derivatives for transposon mutagenesis and for construction of lacZ operon fusions by transposition. Gene.

[68] Ruvkun G.B., Ausubel F.M. (1981). A general method for site-directed mutagenesis in prokaryotes. Nature.

[69] Hayes F. (2003). Transposon-Based Strategies for Microbial Functional Genomics and Proteomics. Annu. Rev. Genet..

[70] Telke A.A., Olaitan A.O., Morand S., Rolain J.M. (2017). SoxRS induces colistin hetero-resistance in Enterobacter asburiae and Enterobacter cloacae by regulating the acrAB-tolC efflux pump. J. Antimicrob. Chemother..

[71] Huang L., Feng Y., Zong Z. (2019). Heterogeneous resistance to colistin in Enterobacter cloacae complex due to a new small transmembrane protein. J. Antimicrob. Chemother..

[72] Lampe D.J., Akerley B.J., Rubin E.J., Mekalanos J.J., Robertson H.M. (1999). Hyperactive transposase mutants of the Himar1 mariner transposon. Proc. Natl. Acad. Sci. USA.

[73] Rubin E.J., Akerley B.J., Novik V.N., Lampe D.J., Husson R.N., Mekalanos J.J. (1999). In vivo transposition of mariner-based elements in enteric bacteria and mycobacteria. Proc. Natl. Acad. Sci. USA.

[74] Crofts T.S., Gasparrini A.J., Dantas G. (2017). Next-generation approaches to understand and combat the antibiotic resistome. Nat. Rev. Microbiol..

[75] Schürch A.C., van Schaik W. (2017). Challenges and opportunities for whole-genome sequencing-based surveillance of antibiotic resistance. Ann. N. Y. Acad. Sci..

[76] Fitzpatrick D., Walsh F. (2016). Antibiotic resistance genes across a wide variety of metagenomes. FEMS Microbiol. Ecol..

[77] Feng Y. (2018). Transferability of MCR-1/2 Polymyxin Resistance: Complex Dissemination and Genetic Mechanism. ACS Infect. Dis..

[78] Khedher M.B., Baron S.A., Riziki T., Ruimy R., Raoult D., Diene S.M., Rolain J.M. (2020). Massive analysis of 64,628 bacterial genomes to decipher water reservoir and origin of mobile colistin resistance genes: Is there another role for these enzymes?. Sci. Rep..

[79] Wang Y., Xu C., Zhang R., Chen Y., Shen Y., Hu F., Liu D., Lu J., Guo Y., Xia X. (2020). Changes in colistin resistance and mcr-1 abundance in Escherichia coli of animal and human origins following the ban of colistin-positive additives in China: An epidemiological comparative study. Lancet Infect. Dis..

[80] Xavier B.B., Lammens C., Ruhal R., Singh K.S., Butaye P., Goossens H., Kumar M.S. (2016). Identification of a novel plasmid-mediated colistin-resistance gene, mcr-2, in Escherichia coli, Belgium, June 2016. Eurosurveillance.

[81] Yin W., Li H., Shen Y., Liu Z., Wang S., Shen Z., Zhang R., Walsh T.R., Shen J., Wang Y. (2017). Novel plasmid-mediated colistin resistance gene mcr-3 in Escherichia coli. MBio.

[82] Carattoli A., Villa L., Feudi C., Curcio L., Orsini S., Luppi A., Pezzotti G., Magistrali C.F. (2017). Novel plasmid-mediated colistin resistance mcr-4 gene in Salmonella and Escherichia coli, Italy 2013, Spain and Belgium, 2015 to 2016. Eurosurveillance.

[83] Borowiak M., Fischer J., Hammerl J.A., Hendriksen R.S., Szabo I., Malorny B. (2017). Identification of a novel transposon-associated phosphoethanolamine transferase gene, mcr-5, conferring colistin resistance in d-tartrate fermenting Salmonella enterica subsp. enterica serovar Paratyphi B. J. Antimicrob. Chemother..

[84] Yang Y.Q., Li Y.X., Lei C.W., Zhang A.Y., Wang H.N. (2018). Novel plasmid-mediated colistin resistance gene mcr-7.1 in Klebsiella pneumoniae. J. Antimicrob. Chemother..

[85] Wang X., Wang Y., Zhou Y., Li J., Yin W., Wang S., Zhang S., Shen J., Shen Z., Wang Y. (2018). Emergence of a novel mobile colistin resistance gene, mcr-8, in NDM-producing Klebsiella pneumoniae. Emerg. Microbes Infect..

[86] Carroll L.M., Gaballa A., Guldimann C., Sullivan G., Henderson L.O., Wiedmann M. (2019). Identification of novel mobilized colistin resistance gene mcr-9 in a multidrug-resistant, colistin-susceptible salmonella enterica serotype typhimurium isolate. MBio.

[87] Wang C., Feng Y., Liu L., Wei L., Kang M., Zong Z. (2020). Identification of novel mobile colistin resistance gene mcr-10. Emerg. Microbes Infect..

[88] Hadjadj L., Baron S.A., Olaitan A.O., Morand S., Rolain J.M. (2019). Co-occurrence of Variants of mcr-3 and mcr-8 Genes in a Klebsiella pneumoniae Isolate from Laos. Front. Microbiol..

[89] Gallardo A., Ruiz U.M., Hernández M., Villoldo M.P., Lázaro R.D., Domínguez L., Quesada A. (2020). Involvement of hpap2 and dgkA Genes in Colistin Resistance Mediated by mcr Determinants. Antibiotics.

[90] Barcz A.K., Gomez J.E., Kaufmann B.B., Hinson E.R., Cosimi L., Borowsky M.L., Onderdonk A.B., Stanley S.A., Kaur D., Bryant K.F. (2012). RNA signatures allow rapid identification of pathogens and antibiotic susceptibilities. Proc. Natl. Acad. Sci. USA.

[91] Dunne W.M., Jaillard M., Rochas O., Van Belkum A. (2017). Microbial genomics and antimicrobial susceptibility testing. Expert Rev. Mol. Diagn..

[92] Guigo R., de Hoon M. (2018). Recent advances in functional genome analysis. F1000Research.

[93] Peng B., Li H., Peng X. (2019). Proteomics approach to understand bacterial antibiotic resistance strategies. Expert Rev. Proteom..

[94] Li H., Wang Y., Meng Q., Wang Y., Xia G., Xia X., Shen J. (2019). Comprehensive proteomic and metabolomic profiling of mcr-1-mediated colistin resistance in Escherichia coli. Int. J. Antimicrob. Agents.

[95] Vranakis I., Goniotakis I., Psaroulaki A., Sandalakis V., Tselentis Y., Gevaert K., Tsiotis G. (2014). Proteome studies of bacterial antibiotic resistance mechanisms. J. Proteomics.

[96] Reyes F.M., Falcón R.M., Chiva C., Pachón J., Andreu D., Rivas L. (2009). The cost of resistance to colistin in Acinetobacter baumannii: A proteomic perspective. Proteomics.

[97] Sun L., Rasmussen P.K., Bai Y., Chen X., Cai T., Wang J., Guo X., Xie Z., Ding X., Niu L. (2020). Proteomic changes of Klebsiella pneumoniae in response to colistin treatment and crrB mutation-mediated colistin resistance. Antimicrob. Agents Chemother..

[98] Luo M.L., Leenay R.T., Beisel C.L. (2016). Current and future prospects for CRISPR-based tools in bacteria. Biotechnol. Bioeng..

[99] Doudna J.A., Charpentier E. (2014). The new frontier of genome engineering with CRISPR-Cas9. Science.

[100] Dong H., Xiang H., Mu D., Wang D., Wang T. (2019). Exploiting a conjugative CRISPR/Cas9 system to eliminate plasmid harbouring the mcr-1 gene from Escherichia coli. Int. J. Antimicrob. Agents.

[101] Sun L., He T., Zhang L., Pang M., Zhang Q., Zhou Y., Bao H., Wang R. (2017). Generation of newly discovered resistance gene mcr-1 knockout in Escherichia coli using the CRISPR/Cas9 system. J. Microbiol. Biotechnol..

[102] Rodrigues M., McBride S.W., Hullahalli K., Palmer K.L., Duerkop B.A. (2019). Conjugative delivery of CRISPR-Cas9 for the selective depletion of antibiotic-resistant enterococci. Antimicrob. Agents Chemother..

[103] Vercoe R.B., Chang J.T., Dy R.L., Taylor C., Gristwood T., Clulow J.S., Richter C., Przybilski R., Pitman A.R., Fineran P.C. (2013). Cytotoxic Chromosomal Targeting by CRISPR/Cas Systems Can Reshape Bacterial Genomes and Expel or Remodel Pathogenicity Islands. PLoS Genet..

[104] Bikard D., Euler C.W., Jiang W., Nussenzweig P.M., Goldberg G.W., Duportet X., Fischetti V.A., Marraffini L.A. (2014). Exploiting CRISPR-cas nucleases to produce sequence-specific antimicrobials. Nat. Biotechnol..

[105] Citorik R.J., Mimee M., Lu T.K. (2014). Sequence-specific antimicrobials using efficiently delivered RNA-guided nucleases. Nat. Biotechnol..

[106] Hao M., He Y., Zhang H., Liao X.P., Liu Y.H., Sun J., Du H., Kreiswirth B.N., Chen L. (2020). CRISPR-Cas9-mediated carbapenemase gene and plasmid curing in carbapenem-resistant enterobacteriaceae. Antimicrob. Agents Chemother..

[107] Kim J.S., Cho D.H., Park M., Chung W.J., Shin D., Ko K.S., Kweon D.H. (2015). Crispr/cas9-mediated re-sensitization of antibiotic-resistant escherichia coli harboring extended-spectrum-lactamases. J. Microbiol. Biotechnol..

[108] Sun Q., Wang Y., Dong N., Shen L., Zhou H., Hu Y., Gu D., Chen S., Zhang R., Ji Q. (2019). Application of CRISPR/Cas9-Based Genome Editing in Studying the Mechanism of Pandrug Resistance in Klebsiella pneumoniae. Antimicrob. Agents Chemother..

[109] McConville T.H., Annavajhala M.K., Giddins M.J., Macesic N., Herrera C.M., Rozenberg F.D., Bhushan G.L., Ahn D., Mancia F., Trent M.S. (2020). CrrB Positively Regulates High-Level Polymyxin Resistance and Virulence in Klebsiella pneumoniae. Cell Rep..

[110] Zhang H., Cheng Q.X., Liu A.M., Zhao G.P., Wang J. (2017). A Novel and Efficient Method for Bacteria Genome Editing Employing both CRISPR/Cas9 and an Antibiotic Resistance Cassette. Front. Microbiol..

